# Sequential Flow–Based Assessment of a Suspected Inferior Pancreaticoduodenal Artery–Derived Pathway Entering the Right Hepatic Artery during Pancreaticoduodenectomy: A Case Report

**DOI:** 10.70352/scrj.cr.26-0551

**Published:** 2026-09-29

**Authors:** Yasutaka Saito, Maharu Furuta, Shohei Sawata, Hirohiko Kuroda, Shinji Hattori

**Affiliations:** Department of Surgery, Masuda Red Cross Hospital, Masuda, Shimane, Japan

**Keywords:** pancreaticoduodenectomy, hepatic arterial inflow, inferior pancreaticoduodenal artery, common hepatic artery, median arcuate ligament, indocyanine green fluorescence imaging

## Abstract

**INTRODUCTION:**

During pancreaticoduodenectomy (PD), hepatic arterial ischemia is usually considered in patients with celiac artery stenosis or occlusion, in whom the gastroduodenal artery (GDA)–based pancreaticoduodenal arcade may serve as a critical collateral pathway. However, a similar but atypical pitfall may exist when the GDA and anterior superior pancreaticoduodenal artery (ASPDA) are absent: a prominent inferior pancreaticoduodenal artery (IPDA)–derived pathway may raise concern regarding hepatic inflow despite the anatomical presence of the common hepatic artery (CHA).

**CASE PRESENTATION:**

A 75-year-old man with distal bile duct cancer underwent preoperative evaluation for PD. Contrast-enhanced CT showed an anatomically identifiable CHA measuring 3.4 mm, whereas the GDA and ASPDA were absent. A markedly developed IPDA-derived arterial pathway coursed cranially from the superior mesenteric artery and appeared to enter the main trunk of the right hepatic artery (RHA) within the hepatoduodenal ligament, raising concern that its division might affect hepatic arterial inflow. Balloon occlusion was performed immediately proximal to the point where the pathway appeared to enter the RHA. During occlusion, aortography showed opacification of the CHA and RHA through the CHA without visually apparent delay. Intraoperatively, temporary clamping with indocyanine green fluorescence imaging showed visually uniform liver surface fluorescence without a subjective right–left difference in fluorescence onset. The pathway was divided, and PD was completed without median arcuate ligament release or arterial reconstruction. Postoperative contrast-enhanced CT on POD 6 confirmed preserved hepatic arterial opacification without ischemic changes.

**CONCLUSIONS:**

An anatomically identifiable CHA does not necessarily guarantee secure hepatic arterial inflow during PD. When the GDA and ASPDA are absent and an IPDA-derived pathway is markedly developed, functional assessment should be considered before irreversible arterial division.

## Abbreviations

ASPDA
: anterior superior pancreaticoduodenal artery
CHA
: common hepatic artery
GDA
: gastroduodenal artery
ICG
: indocyanine green
IPDA
: inferior pancreaticoduodenal artery
MAL
: median arcuate ligament
PD
: pancreaticoduodenectomy
RHA
: right hepatic artery
SMA
: superior mesenteric artery

## INTRODUCTION

Hepatic arterial inflow is a critical consideration during PD. In patients with celiac artery stenosis or occlusion, hepatic perfusion may depend on collateral flow from the SMA through the pancreaticoduodenal arcade and GDA. Therefore, interruption of the GDA-based arcade can result in hepatic ischemia.

However, clinically important hepatic inflow pathways do not always follow this classic pattern. When the GDA and ASPDA are absent, the vessel requiring functional assessment may instead be an IPDA-derived pathway, even when the CHA is anatomically identifiable. We report a case of distal bile duct cancer in which a markedly developed IPDA-to-RHA pathway raised concern for hepatic inflow contribution and prompted preoperative and intraoperative functional assessment before division.

## CASE PRESENTATION

A 75-year-old man was referred to our institution for evaluation and treatment of suspected distal bile duct cancer. He presented with obstructive jaundice and abnormal hepatobiliary enzyme levels. Preoperative laboratory tests showed a total bilirubin level of 4.5 mg/dL, aspartate aminotransferase level of 82 U/L, alanine aminotransferase level of 47 U/L, and gamma-glutamyl transpeptidase level of 478 U/L. Contrast-enhanced CT demonstrated a distal bile duct stricture with wall thickening and enhancement, suspicious for distal bile duct cancer. There was no evidence of distant metastasis or unresectable local invasion.

Preoperative vascular evaluation revealed atypical visceral arterial anatomy. The splenic artery arose separately from the abdominal aorta and was well developed. The CHA was anatomically identifiable and measured approximately 3.4 mm at its proximal segment. The GDA and ASPDA were absent on preoperative CTA, and no GDA or ASPDA trunk was identified intraoperatively. Sagittal CT images showed mild focal narrowing at the origin of the celiac axis with calcified change, but there was no hook-like configuration, post-stenotic dilatation, severe fixed stenosis, or complete occlusion. Clinically significant MAL compression was therefore not evident, although subtle dynamic MAL-related compression could not be entirely excluded.

In contrast, a markedly developed IPDA-derived arterial pathway coursed cranially from the SMA and appeared to enter the main trunk of the RHA within the hepatoduodenal ligament. The combination of the 3.4-mm CHA, absent GDA/ASPDA, and prominent IPDA-derived pathway raised concern that division of the pathway might affect hepatic arterial inflow. Because this pathway would be interrupted during PD, preoperative angiographic flow assessment was performed to determine whether it could be divided safely without arterial reconstruction (**Fig. 1**).

**Fig. 1:**
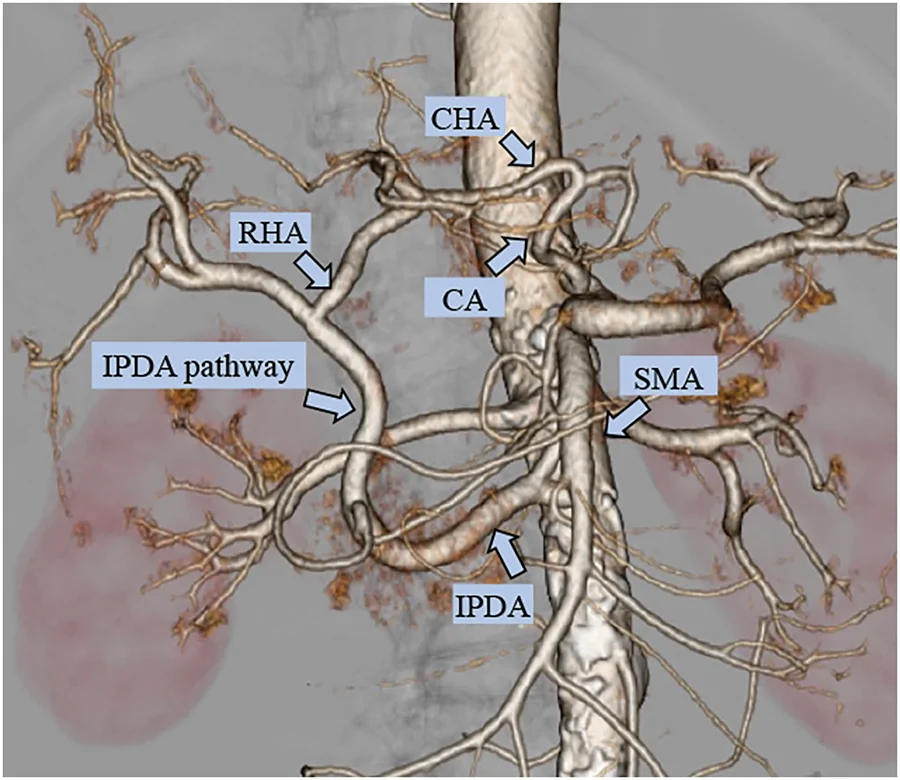
Preoperative 3D CTA. The CHA measured 3.4 mm. The GDA and ASPDA were absent. A markedly developed IPDA-derived pathway coursed cranially from the SMA and appeared to enter the main trunk of the RHA within the hepatoduodenal ligament. ASPDA, anterior superior pancreaticoduodenal artery; CA, celiac artery; CHA, common hepatic artery; GDA, gastroduodenal artery; IPDA, inferior pancreaticoduodenal artery; RHA, right hepatic artery; SMA, superior mesenteric artery

Selective SMA angiography demonstrated an IPDA-derived arterial network with hepatic arterial filling through the pathway toward the RHA. Balloon occlusion was performed immediately proximal to the point where the IPDA-derived pathway appeared to enter the RHA. During balloon occlusion, aortography showed opacification of the CHA and RHA through the CHA without visually apparent delay. These visually assessed findings indicated that hepatic arterial inflow was maintained through the CHA and that the IPDA-derived pathway was not indispensable, supporting PD without planned arterial reconstruction (**Fig. 2**).

**Fig. 2:**
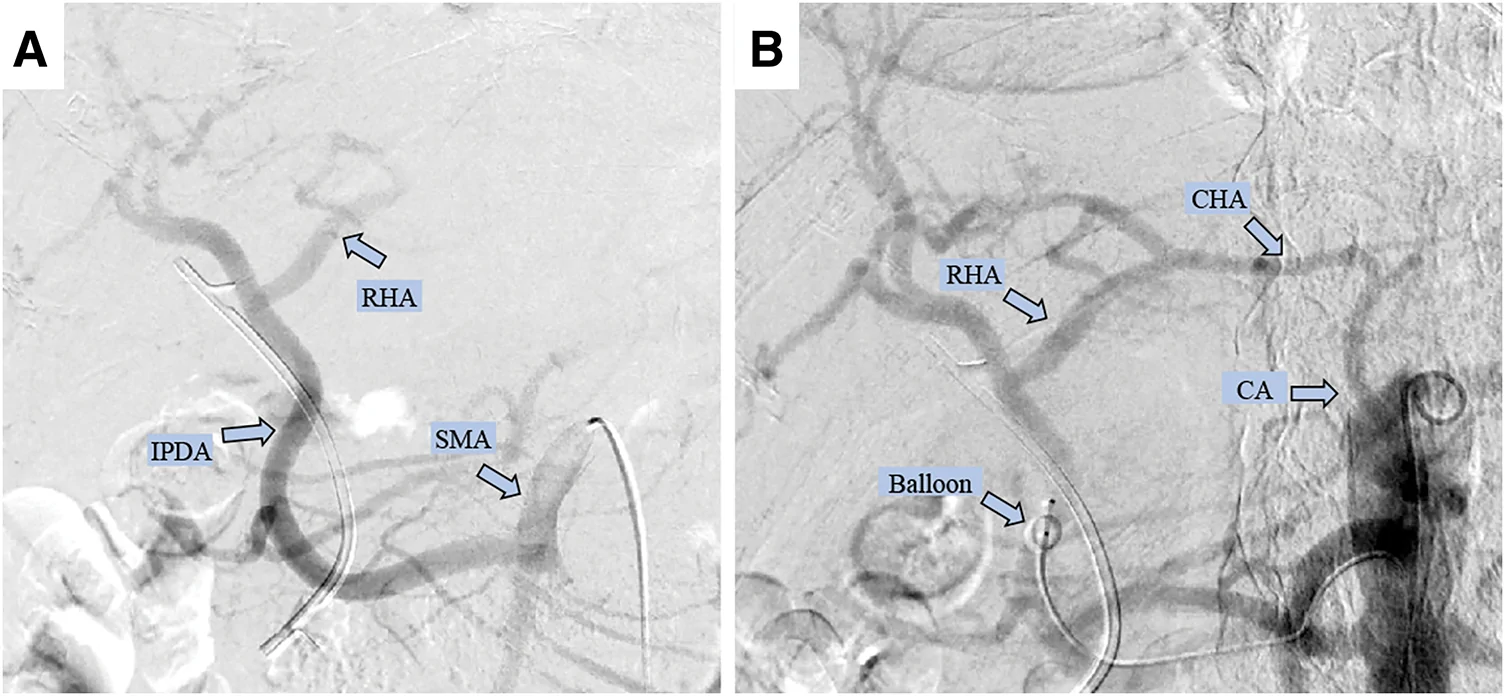
Preoperative angiographic flow assessment. (**A**) SMA angiography demonstrated an IPDA-derived pathway toward the RHA. (**B**) During balloon occlusion immediately proximal to the point where the pathway appeared to enter the RHA, aortography showed opacification of the CHA and RHA through the CHA without visually apparent delay. CA, celiac artery; CHA, common hepatic artery; IPDA, inferior pancreaticoduodenal artery; RHA, right hepatic artery; SMA, superior mesenteric artery

PD was performed through an open approach. Dense adhesions around the hepatoduodenal ligament and pancreatic head made the dissection technically demanding. Additional time was required for careful identification of the atypical arterial anatomy and sequential assessment of hepatic perfusion. No GDA or ASPDA trunk was identified despite careful dissection, consistent with the preoperative finding that these vessels were absent. Before irreversible division of the pancreaticoduodenal arterial network, the suspected IPDA-derived pathway was temporarily clamped. After intravenous administration of ICG, fluorescence imaging showed visually uniform liver surface fluorescence without a segmental defect or a subjective difference in fluorescence onset between the right and left lobes. The pathway was therefore divided, and PD was completed without arterial reconstruction. MAL release was not performed because clinically significant MAL compression was not evident and hepatic perfusion remained adequate during temporary occlusion and clamping. The operative time was approximately 11 h, and the estimated blood loss was approximately 2800 mL. Two units of red blood cells were transfused intraoperatively.

The postoperative course was complicated by a grade B postoperative pancreatic fistula. The drainage catheter was replaced once, after which the fistula improved and the drain was removed. Postoperative laboratory tests showed a total bilirubin level of 1.6 mg/dL, aspartate aminotransferase level of 203 U/L, and alanine aminotransferase level of 133 U/L; all values promptly normalized. There was no clinical or biochemical evidence of hepatic or biliary ischemia. Contrast-enhanced CT on POD 6 confirmed preserved hepatic arterial perfusion without hepatic infarction or ischemic changes (**Fig. 3**). The patient was discharged on POD 32. Final histopathological examination confirmed a 21 × 20-mm tubular adenocarcinoma of the distal bile duct with pancreatic invasion. The pathological classification was pT2N1, with 1 metastatic regional lymph node among 24 examined, and an R0 resection was achieved.

**Fig. 3:**
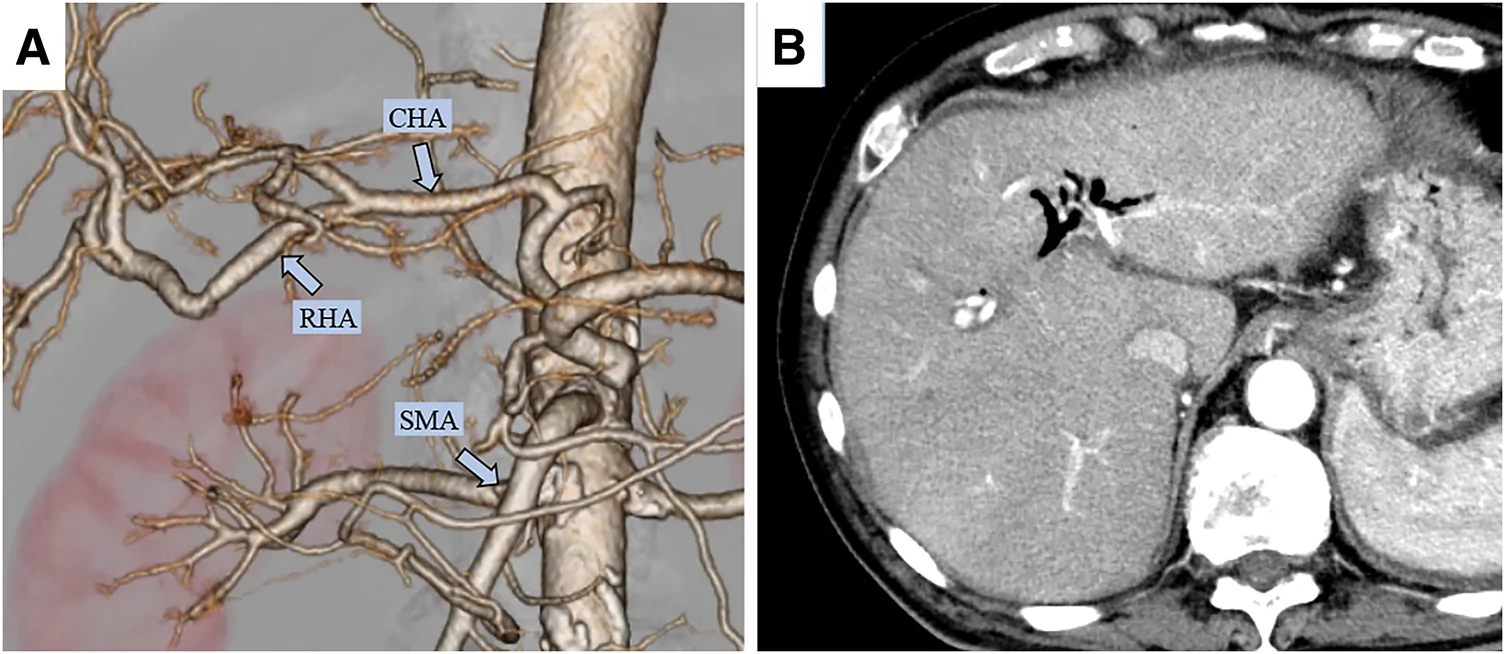
Postoperative imaging on POD 6. (**A**) 3D CTA showed preserved hepatic arterial opacification. (**B**) Contrast-enhanced CT showed no hepatic infarction or ischemic change. CHA, common hepatic artery; RHA, right hepatic artery; SMA, superior mesenteric artery

## DISCUSSION

Hepatic arterial inflow is a critical determinant of safety during PD. Hepatic arterial anatomy is highly variable, and the classic classifications by Michels and Hiatt et al. remain useful for describing replaced or accessory hepatic arteries.^1,2)^ However, these classifications describe anatomical origins and branching patterns and do not necessarily indicate whether a particular arterial pathway is functionally important during PD. In standard anatomy, GDA division is usually tolerated because antegrade hepatic perfusion is maintained through the celiac axis, CHA, and proper hepatic artery. This assumption may fail when celiac-derived hepatic inflow is compromised or when collateral pathways contribute substantially to hepatic perfusion.

The best-recognized situation is celiac artery stenosis or occlusion. The pancreaticoduodenal arterial arcade usually comprises anterior and posterior channels connecting superior pancreaticoduodenal branches from the GDA with inferior pancreaticoduodenal branches from the SMA. The IPDA may arise directly from the SMA or through a common trunk with the first jejunal artery and then divide into anterior and posterior branches. Additional communications through dorsal pancreatic or other peripancreatic branches can link the SMA and celiac circulations.^3)^ In patients with compromised celiac inflow, these channels may enlarge and supply the hepatic artery retrogradely through the GDA. Division of a critical arcade may then cause hepatic or biliary ischemic complications.^4)^ Previous reports have emphasized intraoperative hepatic arterial flow assessment using GDA clamping and Doppler ultrasonography.^5,6)^ In selected patients with complete celiac artery occlusion, preservation of an alternative arterial communication around the pancreatic tail may permit safe GDA division.^7)^

Representative reports relevant to arterial inflow assessment or management during PD are summarized in **Table 1**. The literature includes classic GDA-dependent collateral patterns, combined celiac and hepatic arterial variants, planned hepatic arterial sacrifice, and true IPDA-origin hepatic arterial variants.^5–11)^ The exact incidence of the configuration observed in the present case is unknown. A replaced or accessory RHA arising from the SMA is a recognized hepatic arterial variant, whereas hepatic arterial branches arising from the IPDA or an IPDA-derived pathway toward the RHA have been limited to rare case reports.^1,2,11)^ The present case differed from classic GDA-dependent patterns because the CHA remained patent, the GDA and ASPDA were absent, and the pathway requiring assessment appeared to enter the main RHA within the hepatoduodenal ligament.

**Table 1:** Representative reports relevant to arterial inflow assessment or management during PD

Report	Arterial condition	Pathway or vessel at risk	Assessment/management	Difference from present case
Nara et al.^5)^	Celiac axis stenosis during PD	GDA-based pancreaticoduodenal collateral arcade	Intraoperative GDA clamping and Doppler ultrasonography; arterial reconstruction when hepatic arterial signal decreased	Classic GDA-dependent collateral pattern in celiac axis stenosis
Yamamoto et al.^6)^	MAL-related celiac axis stenosis during PD	GDA-based collateral pathway feeding the hepatic artery	Intraoperative hepatic arterial flow assessment using Doppler ultrasonography and MAL release	MAL compression with classic collateral dependence
Oikawa et al.^7)^	Complete celiac artery occlusion during PD	Alternative arterial communication around the pancreatic tail	Preservation of pancreatic-tail arterial communication allowed GDA division	Celiac artery was completely occluded; the key collateral was around the pancreatic tail
Komatsubara et al.^8)^	Celiac axis stenosis with replaced CHA from SMA during PD	Collateral pathway associated with celiac stenosis and hepatic arterial variation	Surgical management with intraoperative perfusion confirmation	Coexisting celiac stenosis and replaced CHA, unlike the preserved CHA in the present case
Cloyd et al.^9)^	Tumor-involved replaced RHA during PD	Replaced RHA requiring planned sacrifice	Preoperative embolization to promote collateralization before PD	Planned management of a true replaced RHA, not a suspected IPDA-to-RHA flow contribution
Taguchi et al.^10)^	Planned hepatic arterial resection during PPPD	Proper hepatic artery requiring segmental sacrifice	Preoperative segmental embolization to promote collateral flow	Planned hepatic artery sacrifice, not intraoperative division of an IPDA-derived pathway
Sakamoto et al.^11)^	Replaced RHA originating from posterior IPDA during PD	True IPDA-origin replaced RHA	Preoperative identification of aberrant artery and oncologic resection strategy	True arterial branching variant from posterior IPDA
Present case	Preserved 3.4-mm CHA, absent GDA/ASPDA, and prominent IPDA-derived pathway toward RHA	Suspected IPDA-derived pathway entering the main trunk of the RHA within the hepatoduodenal ligament	Preoperative balloon occlusion immediately before RHA entry, followed by intraoperative clamping and ICG fluorescence assessment	Novelty lies primarily in sequential functional assessment of a suspected IPDA-derived RHA pathway despite a preserved CHA

ASPDA, anterior superior pancreaticoduodenal artery; CHA, common hepatic artery; GDA, gastroduodenal artery; ICG, indocyanine green; IPDA, inferior pancreaticoduodenal artery; MAL, median arcuate ligament; PD, pancreaticoduodenectomy; PPPD, pylorus-preserving PD; RHA, right hepatic artery; SMA, superior mesenteric artery

Although the markedly developed IPDA-derived pathway suggested a possible contribution to hepatic arterial inflow, opacification of the CHA and RHA through the CHA without visually apparent delay during balloon occlusion showed that the pathway was not functionally indispensable. The novelty of this case lies not in claiming a previously undescribed arterial variant, but in the sequential functional assessment of a suspected IPDA-derived arterial pathway entering the RHA despite a preserved CHA and absent GDA/ASPDA. The combination of preoperative balloon occlusion and intraoperative clamping with ICG assessment provided clinically useful confirmation before irreversible division.

The mechanism underlying this arterial configuration remains uncertain. Possible explanations include a developmental variation of the pancreaticoduodenal arterial system and structural underdevelopment of celiac-derived branches.^12)^ Mild focal narrowing at the celiac origin with calcified change suggested an atherosclerotic component. Although subtle dynamic MAL-related compression could not be entirely excluded, the absence of a hook-like configuration, post-stenotic dilatation, severe fixed stenosis, or complete occlusion did not support clinically significant MAL compression. Thus, no single congenital or acquired mechanism could be established. A true hepatic arterial branch originating from the posterior IPDA has been reported during PD,^11)^ but that configuration represents a branching variant rather than the suspected flow contribution evaluated in the present case.

Preoperative CTA identified the unusual arterial configuration but could not establish whether the IPDA-derived pathway could be divided safely. Preoperative balloon occlusion provided functional information: aortography showed opacification of the CHA and RHA through the CHA without visually apparent delay. Intraoperative clamping with ICG fluorescence imaging then provided a second, real-time qualitative assessment before irreversible division. ICG findings were based on visual evaluation of liver surface fluorescence, including the subjective comparison of fluorescence onset between the right and left lobes, and were interpreted with the angiographic and anatomical findings rather than as standalone proof of arterial flow. Because both assessments supported preserved perfusion, MAL release and arterial reconstruction were not performed.

Postoperative findings supported this decision. Although the postoperative course was complicated by a grade B pancreatic fistula, there was no biochemical, clinical, or radiological evidence of hepatic ischemia, biliary ischemia, liver abscess, or an ischemic biliary-enteric anastomotic complication. Contrast-enhanced CT on POD 6 confirmed preserved hepatic arterial perfusion without hepatic infarction or ischemic changes.

This case has several limitations. First, the exact embryologic or pathological mechanism underlying the arterial configuration could not be established. Second, balloon occlusion angiography and ICG fluorescence imaging were qualitative. Doppler ultrasonography, pressure or flow measurement, quantitative ICG fluorescence intensity analysis, and time-to-peak analysis were not performed; the balloon occlusion duration was also not recorded. Therefore, the functional assessment was based on visually assessed angiographic opacification, subjective intraoperative liver surface fluorescence, and postoperative contrast-enhanced CT. Third, this was a single case, and the findings should not be generalized to all patients with similar-appearing anatomy. Nevertheless, the case illustrates the importance of functional assessment before arterial division, rather than anatomical prominence alone.

Surgeons should assess not only whether the CHA is present but also whether the GDA and ASPDA are present and which arterial pathway will be interrupted during PD. When the GDA and ASPDA are absent and an IPDA-derived pathway is unusually prominent, preoperative angiographic assessment and intraoperative temporary clamping with perfusion confirmation may help prevent unexpected hepatic ischemia.

## CONCLUSIONS

An anatomically identifiable CHA does not necessarily guarantee secure hepatic arterial inflow during PD. When the GDA and ASPDA are absent and an IPDA-derived pathway is markedly developed, functional assessment should be considered before irreversible arterial division.

## DECLARATIONS

### Funding

The authors received no specific funding for this work.

### Authors’ contributions

YS drafted the manuscript and contributed to patient management.

SH, MF, SS, and HK contributed to patient management and critically revised the manuscript.

All authors reviewed and approved the final manuscript and agree to be accountable for all aspects of the work.

### Availability of data and materials

The data supporting the findings of this report are available from the corresponding author on reasonable request.

### Ethics approval and consent to participate

Ethics approval was waived by the Institutional Review Board of Masuda Red Cross Hospital because this was a single case report.

### Consent for publication

Written informed consent was obtained from the patient for publication of this case report and accompanying images.

### Competing interests

The authors declare that they have no competing interests.
